# Epithelial chemokine CXCL14 synergizes with CXCL12 *via* allosteric modulation of CXCR4

**DOI:** 10.1096/fj.201700013R

**Published:** 2017-03-30

**Authors:** Paul J. Collins, Michelle L. McCully, Laura Martínez-Muñoz, César Santiago, James Wheeldon, Stephan Caucheteux, Sylvia Thelen, Valentina Cecchinato, Julia M. Laufer, Vladimir Purvanov, Yoan R. Monneau, Hugues Lortat-Jacob, Daniel F. Legler, Mariagrazia Uguccioni, Marcus Thelen, Vincent Piguet, Mario Mellado, Bernhard Moser

**Affiliations:** * Division of Infection and Immunity, Cardiff University School of Medicine, Cardiff, United Kingdom;; † Department Immunology and Oncology, Centro Nacional de Biotecnología/Consejo Superior de Investigaciones Científicas, Madrid, Spain;; ‡ Institute for Research in Biomedicine, Università della Svizzera Italiana, Bellinzona, Switzerland;; § Biotechnology Institute Thurgau at the University of Konstanz, Kreuzlingen, Switzerland;; ¶ Konstanz Research School Chemical Biology, University of Konstanz, Konstanz, Germany;; ‖ Institute de Biologie Structurale, Unité Mixtes de Recherche 5075, University Grenoble Alpes, Centre National de la Recherche Scientifique, Commissariat à l'Énergie Atomique, Grenoble, France;; # Department of Medical Biotechnology and Translational Medicine, University of Milan, Milan, Italy

**Keywords:** CXCR4, signal transduction, synergism, allosteric receptor modulation

## Abstract

The chemokine receptor, CXC chemokine receptor 4 (CXCR4), is selective for CXC chemokine ligand 12 (CXCL12), is broadly expressed in blood and tissue cells, and is essential during embryogenesis and hematopoiesis. CXCL14 is a homeostatic chemokine with unknown receptor selectivity and preferential expression in peripheral tissues. Here, we demonstrate that CXCL14 synergized with CXCL12 in the induction of chemokine responses in primary human lymphoid cells and cell lines that express CXCR4. Combining subactive concentrations of CXCL12 with 100–300 nM CXCL14 resulted in chemotaxis responses that exceeded maximal responses that were obtained with CXCL12 alone. CXCL14 did not activate CXCR4-expressing cells (*i.e.,* failed to trigger chemotaxis and Ca^2+^ mobilization, as well as signaling *via* ERK1/2 and the small GTPase Rac1); however, CXCL14 bound to CXCR4 with high affinity, induced redistribution of cell-surface CXCR4, and enhanced HIV-1 infection by >3-fold. We postulate that CXCL14 is a positive allosteric modulator of CXCR4 that enhances the potency of CXCR4 ligands. Our findings provide new insights that will inform the development of novel therapeutics that target CXCR4 in a range of diseases, including cancer, autoimmunity, and HIV.—Collins, P. J., McCully, M. L., Martínez-Muñoz, L., Santiago, C., Wheeldon, J., Caucheteux, S., Thelen, S., Cecchinato, V., Laufer, J. M., Purvanov, V., Monneau, Y. R., Lortat-Jacob, H., Legler, D. F., Uguccioni, M., Thelen, M., Piguet, V., Mellado, M., Moser, B. Epithelial chemokine CXCL14 synergizes with CXCL12 *via* allosteric modulation of CXCR4.

Immune cell traffic and tissue localization is controlled by chemokines, a large family of chemotactic cytokines that is composed of approximately 50 members (1–3). The chemokine family can be broadly divided into 2 functional subsets. Inflammatory chemokines are induced locally in response to inflammatory stimuli, where they recruit immune effector cells to the site of infection or injury. In contrast, homeostatic chemokines are constitutively expressed in healthy tissues, where they control the steady-state traffic of immune cells during tissue immune surveillance. Some homeostatic chemokines were found to be expressed in ectopic lymphoid structures associated with tumors and chronically inflamed tissues (4–6), which underscores their dual role in homeostasis and disease; however, the primary function allocated to homeostatic chemokines—as shown for CXC chemokine ligand 13 (CXCL13), CC chemokine ligand 19 (CCL19), and CCL21—is the control of immune cell traffic within secondary lymphoid tissues, such as spleen, lymph nodes, and Peyer’s patches. Another homeostatic chemokine, CXCL12, controls the retention of hematopoietic stem cells in adult bone marrow and also plays a nonredundant role in organ development during embryogenesis (7). CXCL12 also acts as an HIV-1 suppressor factor as it blocks the entry of virus particles that require CXC chemokine receptor 4 (CXCR4) as coreceptor, together with CD4, for entry of target cells (8, 9). The prototype function of homeostatic chemokines occurs in healthy tissues, which renders them more difficult to study in humans where healthy (disease-free) tissue specimens are not routinely available. Body-lining tissues are extremely diverse in terms of histology, reparative capacity, and composition of immune surveillance cells—macrophages, dendritic cells (DCs), T cells, innate lymphocytes and NK cells—and it is safe to assume that local chemokines play an important role in governing tissue health (10, 11).

Such a role in the control of immune surveillance in epithelial tissues, as well as other sites that undergo frequent tissue remodeling, may be fulfilled by CXCL14 (12, 13). We (14–16) and others (17–21) have reported that CXCL14 is ubiquitously and abundantly expressed in various normal epithelial tissues, including the digestive and urinary tract, placenta, tongue, breast, kidney, and skin. CXCL14 production is most frequently associated with epithelial cells and local macrophages (14–16). Its expression is usually down-regulated in inflammatory settings, which indicates that CXCL14 plays an important role in the maintenance of tissue homeostasis (14, 22). Its function has not been fully elucidated; however, candidate target cells, which have been shown to migrate in response to CXCL14, include blood monocytes (14), neutrophils (20), immature DCs (20, 23–25), and NK cells (26, 27). T and B cells, in contrast, do not migrate toward CXCL14. Expression of CXCL14 in diverse types of cancer is variable and its role in tumor progression remains controversial (reviewed in ref. 28). CXCL14-KO mice display a substantial breeding defect, such that the majority of CXCL14-KO neonates do not survive the first 24 h after birth (29–31). Our studies with the few CXCL14-KO mice that did live to adulthood did not reveal gross abnormalities in the number and location of immune surveillance cells (29). More recent studies have correlated CXCL14 expression with recruitment of white adipose tissue macrophages, as well as the development of insulin resistance and aberrant feeding behavior (30, 31). Clearly, definite clarification of the role played by CXCL14 in physiologic and pathophysiologic processes requires the identification of its receptor, which likely belongs to the class of GPCRs (14).

Recently, it has been reported that CXCL14 is a ligand for CXCR4 (32, 33), the specific receptor for CXCL12, although this finding is still controversial (34). Of note, CXCL14 and CXCL12 are considered to be archetypic chemokines on the basis of their cross-species sequence conservation and the presence of their orthologs in lower vertebrates. In fact, both chemokines are expressed constitutively in the developing organs of mouse, chicken, and zebrafish embryos (35–38). In agreement, the CXCL12/CXCR4 axis governs tissue development during embryogenesis such that homozygous deletions in the genes for either CXCL12 or its receptor prevent the development of viable offspring (7, 39). The striking breeding defects observed in CXCL14-KO mice indicate that CXCL14 fulfils an essential role that is similar to CXCL12 in early development and, in addition, may govern certain aspects of immune surveillance in adult peripheral tissues. Here, we report a strong functional synergy between CXCL14 and CXCL12 that was fully dependent on CXCR4 and involved a direct interaction between CXCL14 and CXCR4. CXCL14 on its own did not induce CXCR4 signaling. Instead, the binding of CXCL14 to CXCR4 enhanced the sensitivity of CXCR4-expressing cells for traditional ligands, including CXCL12 and HIV-1 virions.

## MATERIALS AND METHODS

### Chemokines and other reagents

Synthetic chemokines (CXCL14, CCL2, CCL5, full-length CXCL12α, and CXCL12α N-terminal peptide consisting of amino acid residues 1–9) were chemically synthesized, as previously described (40). Human CXCL14 and murine CCL1—both conjugated to the fluorochrome Alexa Fluor 647 attached to a C-terminal lysine residue—were purchased from Almac Sciences (Edinburgh, United Kingdom). For NMR study, ^15^N-labeled CXCL12α was produced recombinantly and purified as previously described (41). The CXCR4 antagonist AMD3100 was purchased from Abcam (Cambridge, United Kingdom).

### Isolation of primary human cells

All research that involved work with human venous blood samples was approved by the local research ethics commission and informed consent was obtained from each participant. Peripheral blood mononuclear cells (PBMCs) were prepared from the heparinized blood of healthy volunteers by centrifugation over a Lymphoprep (Axis Shield, Dundee, United Kingdom) gradient.

### *In vitro* cell cultures

For all cultures we used RPMI-1640 medium that was supplemented with 10% fetal calf serum (FCS), 2 mM l-glutamine, 1 mM sodium pyruvate, 1% nonessential amino acids, and 50 µg/ml penicillin/streptomycin (complete RPMI; all from Thermo Fisher Scientific, Waltham, MA, USA). Murine pre–B-cell line 300.19 was cultured in the same medium that was supplemented with 50 µM 2-ME (Thermo Fisher Scientific). Cells were maintained in a humidified incubator at 37°C and a mixture of 95% air, 5% CO_2_. 300.19 cells have been routinely used by our group and others for stable transfection with chemokine receptors (9). Parental (untransfected) and 300.19 cells that were stably transfected with either CXCR4 or CC chemokine receptor 2 (CCR2) were maintained at a cell density not exceeding 2 × 10^6^ cells/ml. All cell lines were routinely tested for mycoplasma contamination by RT-PCR.

### Transwell chemotaxis assay

PBMCs or 300.19 cells were spun down and resuspended in prewarmed chemotaxis buffer [plain RPMI-1640 that contained 1% pasteurized plasma protein solution (5% PPL SRK; Swiss Red Cross Laboratory, Bern, Switzerland) and 20 mM HEPES (Thermo Fisher Scientific)] at 2 × 10^6^ cells/ml. Cells were allowed to rest for 30 min at 37°C before assay. Chemokine was resuspended in chemotaxis buffer to the desired concentration and 235 µl was placed in the lower chamber of Transwell 96-well plates (4.26 mm, 5.0 µm pore; Corning, St. David’s Park, United Kingdom). A well that contained chemotaxis buffer alone (blank) served as a negative control. Bare polycarbonate filters were placed in wells, and the plate was placed at 37°C to equilibrate. Cells (160,000; 80 µl) were placed in the upper chamber of the Transwell, and the plate was then incubated at 37°C for 2–4 h. Upon termination of the assay, filters were lifted out of the wells, and the volume in the lower chamber that contained migrated cells was transferred to fluorescence-activated cell sorting (FACS) tubes. Cells were washed once in PBS that contained 2% FCS + 0.02% sodium azide (FACS buffer) before being resuspended in 75 µl FACS buffer. Accu-Check (25 µl) counting beads (Thermo Fisher Scientific) were added to each sample to enable absolute cell counts (final volume per sample = 100 µl) determined by flow cytometry. Cell migration is expressed either as a percentage of total input cells or as the chemotactic index, which is defined as the number of cells migrated in response to chemokine divided by the number of cells that migrated in response to buffer alone (blank).

### Flow cytometry

Single-cell suspensions were incubated with AQUA Live/Dead Fixable Dye (Thermo Fisher Scientific) to allow for exclusion of dead cells. After blocking of endogenous Fc receptors, cells were incubated with fluorochrome-conjugated mAbs against the following human cell-surface markers (conjugate and clone indicated in parentheses): CD3 (Brilliant Violet 421, UCHT1), CD19 [phycoerythrin (PE)-Cy5, HIB19], CD56 (PE, HCD56), CXCR4 (Brilliant Violet 421, 12G5), CCR2 (allophycocyanin, K036C2), and CCR5 (PE, J418F1; BioLegend, London, United Kingdom); CD16 (FITC, 3G8; BD Biosciences, Oxford, United Kingdom); and CD14 (PE-Cy7, 61D3) and CD19 (allophycocyanin, SJ25C1; eBioscience, Hatfield, United Kingdom). Staining with Abs was performed in FACS buffer for 30 min at 4°C. Isotype matched control Abs were used as appropriate. Binding of AF647-CXCL14 or AF647-muCCL1 (50 nM) was performed in FACS buffer for 30 min at 4°C in a step before Ab staining. Sample acquisition was performed by using a FACS Canto II instrument (BD Biosciences). Cell aggregates were excluded according to light scatter properties. Data were analyzed by using FlowJo software (ver. 10.04; TreeStar, Ashland, OR, USA).

### [Ca^2+^]_i_ rise

Pre-B 300.19-CXCR4^+^ cells (0.2 × 10^6^) in 200 µl were loaded with FURA 2-AM (final concentration 1 µM) on poly-d-lysine–coated slides (MatTek, Ashland, MA, USA). Loaded cells were washed with buffer that contained 136 mM NaCl, 4.8 mM KCl, 20 mM HEPES, 1 mM CaCl_2_, and 10 mM glucose. Imaging was recorded with a ×40 oil-immersion objective on an inverted microscope (Axiovert 200; Carl Zeiss, Jena, Germany) with excitation at 340 and 380 nm using the Polychrom V illumination system from Till Photonics GmbH (Munich, Germany). Chemokine was injected after 50 s of recording, and recording continued up to 300 s. The 340/380 ratio provides a relative measure of cytoplasmic free Ca^2+^ concentration.

### ERK phosphorylation and Rac1 activation

Pre-B 300.19-CXCR4^+^ cells (10^6^/ml) were starved in complete RPMI without FCS for 8 h, then stimulated with CXCL12 ± CXCL14 for 2, 10, or 20 min at 37°C in a total volume of 200 µl. Reaction was stopped by addition of 200 µl 20% TFA and phosphorylated ERK was determined (42). For Rac1 activation studies, human peripheral blood CD3^+^ T cells, either untreated or treated with inhibitors (2 h with 40 µM PP2 or 15 min with 10 µM AMD3100), were stimulated for 3 min with the indicated concentrations of CXCL12 and/or CXCL14. Cells were then fixed in 4% paraformaldehyde, stained for intracellular active Rac1 (Rac1-GTP), and analyzed by flow cytometry or confocal microscopy.

### NMR experiments

All NMR experiments were performed on a Bruker 850-MHz spectrometer equipped with a CryoProbe (Bruker, Billerica, MA, USA). The putative interaction of CXCL14 with CXCL12 was investigated by comparing the [^15^N,^1^H]-HSQC spectra recorded on ^15^N-labeled CXCL12 at 77 μM (dissolved in 20 mM phosphate buffer, 100 mM NaCl, pH 6), either alone or mixed with an equimolar concentration of unlabeled CXCL14 (dissolved in standard PBS). The combined chemical shift perturbation of the *i*^th^ residue of CXCL12 upon CXCL14 addition was calculated by using the following equation (43):that resulted in 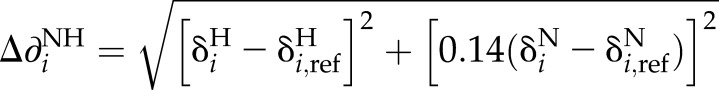
with 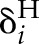 and 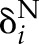, the ^1^H and ^15^N chemical shifts, respectively, of the *i*^th^ residue; 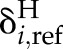 and 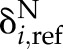, the ^1^H and ^15^N chemical shifts, respectively, of the *i*^th^ residue in the reference spectrum.

### Surface plasmon resonance experiments

For CXCL14 and CXCL12 binding analysis to CXCR4, we produced and characterized lentiviral particles (LVPs) by cotransfection of HEK293T cells that overexpressed CXCR4 (44). LVPs were analyzed for CXCR4 expression by flow cytometry (44). Several batches of LVPs were standardized for CXCR4 expression, aliquoted, and stored at −80°C. Surface plasmon resonance (SPR) experiments were performed on a Biacore 3000 (GE Healthcare, Pittsburgh, PA, USA). Flow cells of a CM5 sensorchip were functionalized with 8000–8500 resonance units of LVP as described in Vega *et al*. (44). CXCL12 or CXCL14 (12.5–200 nM) diluted in HBS buffer (100 mM HEPES, 150 mM NaCl, 0.005% polyoxyethylene sorbitan P20, pH 7.4) were injected over immobilized LVPs (30 μl/min, 2 min, 25°C; association phase), followed by a 4-min injection period of HBS buffer alone over the surface (dissociation phase). Sensorgrams were corrected for signals obtained in reference flow channels (sensorchips that contained immobilized LVPs obtained from CXCR4-negative HEK293T cells or no LVPs). Sensorchip regeneration involved washing with 5 mM HCl. All steps were performed by using system robotics. Sensorgrams were recorded in real time and expressed in relative units. Sensorgrams were used to calculate (simple 1-site interaction model with Biaevaluation 4.1 software; Biacore) kinetic parameters.

### Förster resonance energy transfer experiments

HEK293T cells (9 × 10^6^ cells) were transiently cotransfected with 9 µg CXCR4–cyan fluorescent protein (CFP) and 15 µg CXCR4–yellow fluorescent protein (YFP) for assays at fixed ratios. After 48 h, cells were washed twice in HBSS that was supplemented with 0.1% glucose and distributed into black 96-well microplates for fluorescence measurements (Wallac Envision 2104 Multilabel Reader; PerkinElmer, Waltham, MA, USA). To calculate Förster resonance energy transfer (FRET) efficiency, we separated the relative contribution of fluorophores to the detection channels for linear unmixing (45). When required, cells were stimulated with ligands for 30 min at 37°C. To determine the fluorescence emitted by each fluorophore, we applied the following formulas: CFP = S/(1 + 1/R) and YFP = S/1 + R, where S = ChCFP + ChYFP; R = (YFP_530_Q – YFP_510_)/(CFP_510_ – CFP_530_Q); and Q = ChCFP/ChYFP. ChCFP and ChYFP represent the signal in the 510- and 530-nm detection channels (Ch); CFP_510_, CFP_530_, YFP_530_, and YFP_510_ represent the normalized contributions of CFP and YFP to channels 510–530, as determined from spectral signatures of fluorescent proteins. Results were analyzed by using a 1-way ANOVA followed by Kruskall-Wallis test and a posttest of Dunn’s multiple comparison. We used GraphPad Prism 5.0 (GraphPad Software, La Jolla, CA, USA).

### *In vitro* HIV-1 infection assays

In the HIV-1 luciferase assay, TZM-bl cells were pretreated with either CXCL12 (0–1000 nM), CXCL14 (0–1000 nM), or CXCL14 + CXCL12 (0.1 nM) for 1 h before infection. Cells were infected with 3 ng of either R8-Bal R5-tropic or pNL4.3 X4-tropic HIV-1 virions for 3 h, washed, and cultured in 100 µl chemokine-containing medium for 48 h. After culture, 100 µl SteadyGlo reagent (E2510; Promega, Madison, WI, USA) was added to wells and allowed 5 min to lyse cells before being analyzed on a luminometer. In the HIV-1 GHOST-R5 infection assay, GHOST-R5 (AIDS Reagent Program, Frederick, MD, USA) cells were pretreated 1 h before infection with CCL5/CXCL14 (0–1000 nM), spinoculated with 250 pg R8-Bal R5-tropic HIV-1 for 2.5 h at 37°C, washed with 1× PBS, and cultured for 48 h at 37°C in chemokine-containing medium. Cells were fixed in 4% paraformaldehyde for 30 min, stained for intracellular p24 HIV-1 core antigen (RD1, 6604667, Coulter Clone; Beckman Coulter, Brea, CA, USA), and analyzed by using flow cytometry (FACS Canto II; BD Biosciences).

### Statistical analyses

For chemotaxis assays, statistical significance was determined by using a 1-way ANOVA followed by Bonferroni *post hoc* testing. Results were considered significant at *P* < 0.05, *P* < 0.01, or *P* < 0.001. Statistical analyses were performed by using Prism, ver. 6.0 (GraphPad Software).

## RESULTS

### Direct binding of CXCL14 to CXCR4

We first set out to readdress the CXCL14 binding studies reported by Tanegashima and colleagues (32, 33), which were not supported by functional studies carried out by another group (34). We have developed an SPR-based binding assay that can be applied to measuring the on- and off-rates, in real-time, of chemokines interacting with their receptors (44). In brief, CXCR4-expressing LVPs are immobilized on an SPR biosensor chip, and SPR signals are recorded after injection of chemokine. In agreement with previous results (44), we determined the on-rate (*k*_on_ = 1.22 × 10^5^ M^−1^ · s^−1^) and off-rate (*k*_off_ = 4.28 × 10^−3^ s^−1^) of CXCL12 interaction with CXCR4, which resulted in a dissociation constant (*K*_D_) of 3.5 × 10^−8^ M. Substituting CXCL12 with CXCL14 revealed strong binding of CXCL14 for immobilized CXCR4 with on-rate (*k*_on_ = 3.96 × 10^5^ M^−1^ · s^−1^) and off-rate (*k*_off_ = 3.17 x 10^−3^ s^−1^) that resulted in a *K*_D_ of 8.0 × 10^−9^ M (Supplemental Fig. 1*A*), which is similar to the published *K*_D_ value of 1.47 × 10^−8^ M obtained by Tanegashima's group using [^125^I]-CXCL14 (32). In addition, CXCL14 efficiently displaced bound CXCL12 as assessed by the sequential injection of increasing concentrations of CXCL14 to SPR sensor chips that were pretreated with a single concentration of CXCL12 (Supplemental Fig. 1*B*). In the reverse experiment, CXCL12 was consistently less efficient in displacing bound CXCL14. We conclude that CXCL14, indeed, is a ligand for CXCR4; however, as suggested by displacement binding experiments, the 2 chemokines differ in the way they interact with CXCR4 which may be explained by distinct and, in part, nonoverlapping binding sites on the receptor.

### CXCL14 synergizes with CXCL12 in mediating CXCR4-dependent cell migration

**Figure 1.**
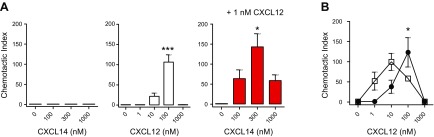
CXCL14 synergizes with CXCL12 in the induction of chemotactic migration of CXCR4-expressing cells. *A*) Chemotactic migration of 300.19 CXCR4^+^ cells toward CXCL14 (black bars), CXCL12 (white bars), or CXCL14 (100–1000 nM) in combination with a fixed concentration (1 nM) of CXCL12 (red bars). Migration is expressed as chemotactic index and means + sem of 2–8 independent experiments. *P < 0.05, ***P < 0.001 compared with migration in the absence of chemokine (0 nM; 1-way ANOVA plus Bonferroni posttest). *B*) Migration of 300.19 CXCR4^+^ cells toward CXCL12 alone (filled circles) or CXCL12 plus a fixed concentration (300 nM) of CXCL14 (open squares); mean ± sem of 3 independent experiments is shown. *P < 0.05 (100 nM CXCL12 *vs*. 100 nM CXCL12 + 300 nM CXCL14); 2-way ANOVA plus Bonferroni posttest.

We next examined the ability of CXCL14 to induce chemokine-typical responses in the mouse pre–B-cell line 300-19 that was stably transfected with CXCR4. CXCL14 was completely inactive up to a concentration 1000 nM in inducing chemotactic migration, whereas CXCL12 was active with maximal migratory responses observed at 100 nM (**Fig. 1*A***). We expected that CXCL14 may inhibit CXCL12 activity, but to our surprise, we observed a strong cooperativity between CXCL14 and CXCL12. Combination of a low (subactive) concentration of CXCL12 [0.1 nM (not shown) or 1 nM], which by itself does not induce chemotaxis, with CXCL14 resulted in a strong migratory response that peaked at 300 nM CXCL14. The response to 1 nM CXCL12 + 300 nM CXCL14 even exceeded maximal responses obtained with 100 nM CXCL12 alone (Fig. 1*A*). In the presence of CXCL14, CXCL12 exhibited >10-fold enhanced potency in the migration response (Fig. 1*B*).

**Figure 2.**
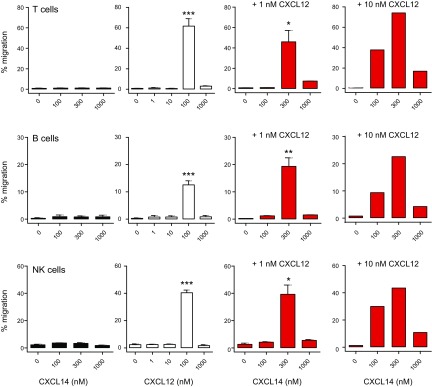
CXCL14 synergizes with CXCL12 in chemotaxis of primary blood lymphocytes and NK cells. Chemotactic responses of freshly isolated human PBMCs toward CXCL14 (black bars), CXCL12 (white bars), or CXCL14 (100–1000 nM) plus a fixed concentration (1 or 10 nM) of CXCL12 (red bars). Input and migrated cells were counted by flow cytometry, with gating on surface markers CD3, CD19, and CD56 to distinguish T cells (top row), B cells (middle row), and NK cells (bottom row), respectively. Migration is expressed as a percentage of input of each cell type and means + sem of 3 independent experiments. *P < 0.05, **P < 0.01, ***P < 0.001 compared with all other groups (1-way ANOVA plus Bonferroni posttest).

**Figure 3.**
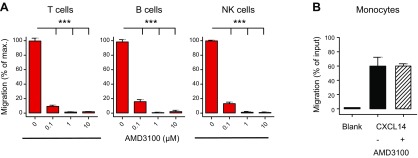
Synergy between CXCL14 and CXCL12 is completely abolished by blockade of CXCR4. PBMCs were pretreated with 0–10 μM AMD3100 before use in chemotaxis assays, as described above. *A*) Migration of T, B, and NK cells in response to the combination of 1 nM CXCL12 plus 300 nM CXCL14 is shown. Migration of each cell type in the absence of AMD3100 treatment (0 µM) is given as 100%. Data are means + sem of 4 blood donors across 2 independent experiments. ***P < 0.001 compared with no AMD3100 treatment, using a 1-way ANOVA plus Bonferroni posttest. *B*) Migration of monocytes toward 1 µM CXCL14 following either no treatment (−) or pretreatment with 10 µM AMD3100 (+). Migration is given as percent of total input monocytes.

CXCR4 is widely expressed in primary cells, including tissue cells and PBMCs; therefore, we tested the synergistic activity between CXCL14 and CXCL12 in freshly isolated T, B, and NK cells that uniformly expressed CXCR4 but did not respond to CXCL14 alone (**Fig. 2**). Lymphocytes were functionally competent, as evidenced by their robust chemotaxis responses to CXCL12, consistently displaying maximal responses at 100 nM CXCL12 (Fig. 2). Of note, the combination of a subactive concentration [0.1 nM (not shown) or 1 nM] of CXCL12 with increasing concentrations of CXCL14 resulted in robust cell migration that peaked at 300 nM CXCL14, which compares well with our results with CXCR4-transfected cells (Fig. 1). As typically observed with chemoattractants, CXCL14 became inhibitory at high (>300 nM) concentrations. The robustness of the observed synergy between these 2 primordial chemokines prompted us to investigate this phenomenon in greater detail. To confirm that the synergy between CXCL14 and CXCL12 occurred *via* CXCR4, the primary receptor for CXCL12 (8, 9), we performed the same experiments by using PBMCs that were pretreated with the CXCR4-specific antagonist, AMD3100 (46). Similar to CXCL12 alone (not shown), T-, B-, and NK-cell migration in response to 1 nM CXCL12 + 300 nM CXCL14 was completely abrogated after pretreatment with 1–10 μM AMD3100, which indicated that the synergy response did indeed depend on CXCR4 (**Fig. 3*A***). CXCL14 is highly selective for blood monocytes (14), and, in fact, recent examinations revealed that the CD14^hi^ subset of primary human monocytes is the major target (not shown). Of interest, migration of monocytes toward CXCL14 alone was not affected by pretreatment with AMD3100 (Fig. 3*B*), which suggested that monocyte responses toward CXCL14 are mediated by a receptor that is unrelated to CXCR4.

### CXCL14 enhanced CXCL12-mediated signal transduction in CXCR4-expressing cells

**Figure 4.**
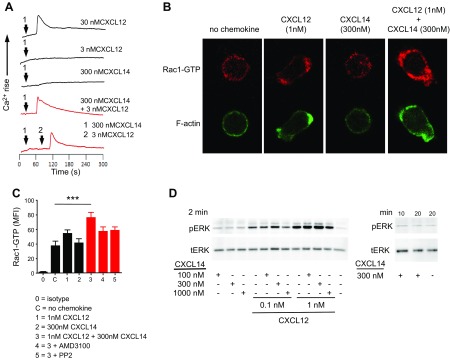
CXCL14 synergized with CXCL12 in Ca^2+^ mobilization and Rac1 activation responses but not in ERK1/2 phosphorylation responses. *A*) Real-time changes in [Ca^2+^]_i_ concentrations in CXCR4-transfected cells in response to indicated chemokines. All experiments were performed at least twice. *B*) Polarization of primary T cells was visualized by staining of F-actin and Rac1-GTP followed by confocal microscopy. *C*) Rac1 activation was measured in flow cytometry by determining mean fluorescent intensity of Rac1-GTP staining in primary T cells by using an Ab that recognizes active GTP-bound Rac1. For each analysis, 10,000 cells were measured. Data are means + sem of 5 experiments derived from individual donors. *D*) CXCR4-transfected cells were treated with chemokines as shown and then processed for Western blot analysis. ERK1/2 phosphorylation was determined with a phospho-ERK1/2–specific Ab. pERK, phosphorylated ERK1/2; tERK, total ERK1/2 protein. Data are representative of 4 experiments with similar results. ***P < 0.001.

Chemokines—upon activation of their corresponding receptor—induce intracellular signaling cascades that result in cell activation and motility that includes G-protein–mediated and –independent pathways (47, 48). As expected from its failure to induce chemotaxis, CXCL14 alone did not induce transient elevations of intracellular Ca^2+^ concentrations, which are typically observed in chemokine receptors coupling to G_αi_ proteins, whereas CXCL12 alone showed expected response profiles (**Fig. 4*A***). Again, combination of 0.5 nM (not shown) or 3 nM CXCL12 (concentrations below the threshold of Ca^2+^ mobilization) with 300 nM CXCL14 resulted in clear and transient Ca^2+^ spikes, even after sequential addition of the 2 chemokines, which indicated that CXCL14 synergized with CXCL12 in a prototypic G_αi_-protein–mediated signaling event.

Small GTPases, including Rho, Cdc42, and Rac isoforms, control cell polarization in the front and rear (uropod), which is a prerequisite for cell migration (48). Primary human T cells that were exposed to 1 nM CXCL12 already induced T-cell polarization as evidenced by F-actin staining (Fig. 4*B*), despite the fact that this concentration was below the threshold for *in vitro* chemotaxis (Figs. 1 and 2). By contrast, 300 nM CXCL14 did not induce T-cell polarization and did not inhibit this response when added together with 1 nM CXCL12 (Fig. 4*B*). Synergistic responses between CXCL14 and CXCL12 in T-cell polarization were not significant. In addition, 1 nM CXCL12 or 300 nM CXCL14 alone did not induce significant GTPase Rac1 activation in primary T cells as measured by flow cytometry with an Ab that specifically recognized active GTP-bound Rac1 (Fig. 4*C*). Of note and in agreement with chemotaxis and Ca^2+^ mobilization responses, synergy between 1 nM CXCL12 and 300 nM CXCL14 resulted in significant elevation in intracellular Rac1-GTP, which was partially blocked by CXCR4 inhibitor, AMD3100, and Src-family kinase inhibitor, PP2 (Fig. 4*C*).

Finally, because of its prominent contribution to biased (G-protein–independent) agonism (49), we examined the MAPK/ERK signaling pathway by Western blot. Stimulation of CXCR4-transfected pre–B cells with 100–1000 nM CXCL14 alone over short (2 min) or long (10 and 20 min) periods of time did not induce pERK generation (Fig. 4*D*). CXCL12, in contrast, generated strong pERK signals, which was in keeping with previous results (50). The combination of 300 nM CXCL14 with either 0.1 or 1 nM CXCL12 induced a mild but consistent synergism that did not exceed 2-fold compared with 0.1 or 1 nM CXCL12 alone. Of note, excess (1000 nM) CXCL14 significantly reduced maximal pERK responses, which resembled synergistic cell migration responses observed at the highest CXCL14 concentrations (Figs. 1 and 2). Collectively, we have demonstrated that CXCL14 alone did not activate CXCR4-associated signaling events, nor did it inhibit CXCL12-mediated responses. Instead, CXCL14 synergized to various degrees with CXCL12 in CXCR4-mediated intracellular signal transduction.

### Mechanism underlying observed CXCL14-CXCL12 synergism

Heterodimerization has been identified as a major mechanism that underlies the synergistic activity of various chemokine pairs (51, 52), as well as chemokines with other inflammatory mediators (53). By comparing 2-dimensional ^15^N[^1^H] chemical shift correlation NMR spectra of ^15^N-labeled proteins before and after addition of partner proteins, it is possible to confirm protein–protein interactions, even between low-affinity binding partners. Furthermore, it is also possible to deduce which residues are involved in the observed interactions (54). We have previously applied this technique to demonstrate that high mobility group box 1 protein (HMGB1) and CXCL12 synergized in the recruitment of immune cells by means of a direct physical interaction (53). Here, however, our recordings of [^15^N,^1^H]-HSQC spectra of ^15^N-labeled CXCL12 and CXCL14 did not reveal such CXCL12–CXCL14 protein interactions, even at very high concentrations (77 μM/each; Supplemental Fig. 2*A*). We next used SPR biosensor measurements in which soluble CXCL12 or CXCL14 were flowed over CXCL14 or CXCL12 immobilized on biosensor chips. In agreement with the above-described results, no significant interactions were revealed (Supplemental Fig. 2*B*), whereas in control experiments, both chemokines bound strongly to immobilized heparan sulfate (Supplemental Fig. 2*C*).

**Figure 5.**
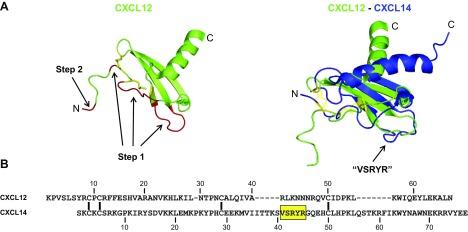
Primary and tertiary structure comparison between CXCL14 and CXCL12. *A*) Ribbon diagrams of CXCL12 alone (left; UniProt P48061) and in combination with CXCL14 (right; UniProt O95715). Step 1 and step 2 indicate regions that are shown to be involved in binding to CXCR4 (step 1) and induction of signal transduction (step 2). Diagrams were computed by using PyMOL. *B*) Single amino acid sequence alignment of mature human CXCL12 and CXCL14. Position of conserved Cys residues are shown by vertical bars; yellow box highlights the extended 40s-loop region in CXCL14.

In addition to the overall diversity in primary amino acid sequences (23.4% sequence identity), CXCL14 and CXCL12 differ fundamentally in 3 regions of their tertiary structures (**Fig. 5*A***). Their respective C-terminal helices do not align; however, as this region in chemokines does not directly interact with cognate receptors, this structural difference is unlikely to explain the observed CXCL14–CXCL12 synergism. Compared with CXCL12 and all other chemokines, the 40s loop that connects the β2 and β3 strands in the β-pleated sheet of CXCL14 contains an extra 5-aa (VSRYR) insertion that may affect the selectivity of CXCL14 for its cognate (as yet unknown) receptor. The most noticeable difference, however, lies in their N-terminal sequences that precede the first of 4 conserved Cys residues (Fig. 5*A*, *B*). The N terminus in CXCL14 is restricted to 2 aa (Ser-Lys), whereas in CXCL12 it is composed of 8 aa that are critical for CXCL12 activity (55). Indeed, residues in the unstructured N terminus of CXCL12 were proposed to bind to the minor binding groove (site 2) within CXCR4, which enabled step 2 in the 2-step binding model that leads to G-protein activation (Fig. 5*A*) (56, 57). Of interest, N-terminal peptides of CXCL12 were able to trigger responses in CXCR4-expressing cells at ≥10 μM concentrations (40). As CXCL14 is missing an equivalent N terminus, we reasoned that CXCL14 binding to CXCR4 would primarily occupy site 1 on CXCR4 while leaving site 2 free for binding of a different ligand. Synthetic CXCL12 (N-terminal residues 1–9) peptide induced chemotaxis in CXCR4-transfected pre–B cells at micromolar concentrations; however, synergy between CXCL14 and CXCL12(1–9) peptide was not observed (Supplemental Fig. 3).

**Figure 6.**
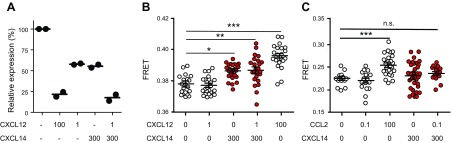
CXCL14 induces redistribution and conformational changes of cell surface CXCR4. *A*) Internalization of CXCR4 after incubation of 300.19 CXCR4^+^ cells with chemokines was determined by flow cytometry. Data from 2 independent experiments are shown; 100% CXCR4 expression refers to staining of untreated 300.19 CXCR4^+^ cells with anti-CXCR4 [mean fluorescence intensity (MFI), 9977 ± 1235), whereas 0% CXCR4 expression refers to staining of parental (untransfected) 300.19 cells (MFI, 148 ± 17). *B*) HEK293T cells were transiently cotransfected at fixed ratio with CXCR4-CFP and CXCR4-YFP to determine the effect of CXCL12 (1 or 100 nM), 300 nM CXCL14, and 1 nM CXCL12 + 300 nM CXCL14. Cells were treated with chemokines (or PBS as negative control) for 30 min at 37°C. *C*) HEK293T cells were transiently cotransfected with CCR2-CFP and CCR2-YFP at a fixed ratio. Cells were treated with CCL2 (0.1 or 100 nM), 300 nM CXCL14, or 0.1 nM CCL2 + 300 nM CXCL14 for 30 min at 37°C, and FRET efficiency was determined as in panel *B*. For data in panels *B* and *C*, statistics were calculated by using a 1-way ANOVA plus nonparametric Kruskall-Wallis test and Dunn’s multiple comparison test. N.s., not significant. *P < 0.05, **P < 0.01, ***P < 0.001.

Although we have demonstrated that binding of CXCL14 to CXCR4 does not trigger chemotaxis and other receptor signaling events, binding of CXCL14 to CXCR4 could modulate the distribution of CXCR4 on the cell surface. Incubation with 300 nM CXCL14 at 37°C led to a modest (approximately 50%) but clear cell-surface depletion of CXCR4, which was equivalent to the receptor internalization obtained with 1 nM CXCL12 (**Fig. 6*A***). Incubation with 300 nM CXCL14 and 1 nM CXCL12 simultaneously depleted surface CXCR4 levels even further (>75%) and was comparable to the effect observed with 100 nM CXCL12 (the optimal concentration required for induction of chemotaxis; Figs. 1 and 2). It has been demonstrated that the clustering of receptors at the cell surface precedes clathrin-mediated receptor endocytosis; thus, we carried out FRET experiments by using HEK293T cells that were transiently transfected with CXCR4 fused to the fluorescence tags, CFP, and YFP. This powerful technique has been previously applied to the study of cell-surface interactions between chemokine receptors, revealing the dynamic formation of homo- and heterodimers and the conformational changes that are triggered by their ligands (45). To set up FRET experiments, receptor transduction conditions that yielded optimal CXCR4-YFP/CXCR4-CFP ratios were determined (Supplemental Fig. 4*A*). In agreement with previous results (58), we detected positive FRET signals in the absence of ligands, which indicated the presence of CXCR4 homodimers under nonactivated conditions. These base-level FRET signals increased when cells were stimulated with 100 nM CXCL12, whereas 1 nM CXCL12 was inactive (Fig. 6*B*). Of interest, 300 nM CXCL14 also promoted significant increase of FRET signals, which was not further enhanced by the addition of 1 nM CXCL12. In control experiments with CCR2-YFP/CCR2-CFP–expressing HEK293T cells (Supplemental Fig. 4*B*), CXCL14 did not modify basal FRET efficiency when added alone or in combination with CCL2, which confirmed the specificity of CXCL14 for CXCR4 (Fig. 6*C*). In agreement, we did not detect synergistic interactions between CXCL14 and CCL2 in the induction of chemotactic responses in CCR2-transfected 300.19 cells (not shown). We conclude that the observed functional synergism between CXCL14 and CXCL12 correlates with CXCL14-induced changes in the conformation and/or dimer formation of cell-surface CXCR4.

### Modulation of HIV-1 infection by CXCL14

**Figure 7.**
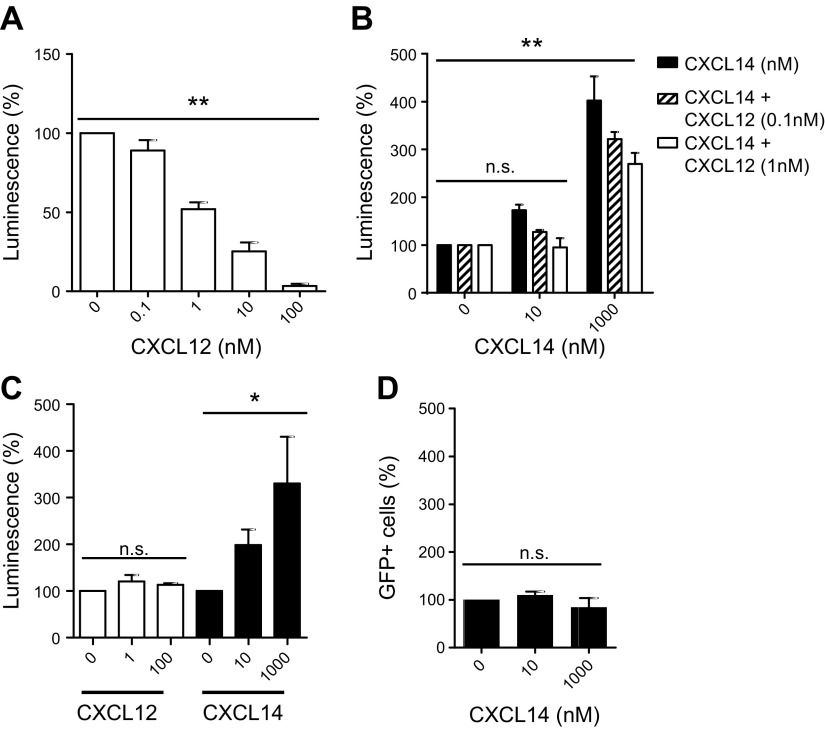
Synergistic effect of CXCL14 on HIV-1 infection. *A*) Entry of CXCR4-tropic HIV-1 particles pNL4.3 into TZM-bl cells that coexpressed CD4, CXCR4, and CCR5 in the presence of increasing concentrations of CXCL12. Viral infection is represented by expression of a luciferase reporter and is normalized to infection in the absence of CXCL12 (medium only = 100% infection). Data are means + sem of 3 independent experiments. *P < 0.05, **P < 0.01 compared with 0 nM CXCL12 (1-way ANOVA plus Bonferroni posttest). *B*) Entry of pNL4.3 into TZM-bl cells in the presence of 0–1000 nM CXCL14 alone (black bars) or CXCL14 in combination with 0.1 nM CXCL12 (open bars); 100% luminescence corresponds to luciferase reporter activity in the absence of chemokines. Data are means + sem of 3 independent experiments. *C*) Entry of CCR5-tropic HIV-1 particles pR8Bal into TZM-bl cells in the presence of 0–100 nM CXCL12 or 0–1000 nM CXCL14 as indicated. Data are means + sem of 3 independent experiments. *D*) Entry of pR8Bal into GHOST cells that coexpress CD4 and CCR5 (but not CXCR4) as well as green fluorescent protein (GFP) under the control of viral long terminal repeat promoter; 100% GFP^+^ cells refers to fluorescence signals obtained after viral infection of GHOST cells in the absence of chemokines. Ns, not significant. Data are means + sem of 3 independent experiments.

Having established that CXCL14 synergizes with CXCL12 by means of direct interaction with CXCR4, we decided to investigate the influence of CXCL14 on HIV infection. Envelope glycoprotein gp120 of HIV-1 is the second known natural ligand of CXCR4. HIV-1 entry requires CD4, as main receptor, in addition to either one of the 2 coreceptors, CXCR4 and CCR5, on target cells, and viral species are defined by their coreceptor usage (*i.e.,* X4-tropic and R5-tropic viruses infect CD4^+^ target cells that coexpress CXCR4 and CCR5, respectively) (59). Chemokines that bind to either CXCR4 or CCR5 were shown to prevent infection by X4 and R5 HIV-1 particles, respectively, and, hence, are referred to as HIV suppressor factors (59). In an *in vitro* single-round HIV-1 entry assay that employs a luciferase reporter readout as a measure of viral entry into the HeLa-derived target cell line TZM-bl, CXCL12, as expected (8, 9), had a potent inhibitory effect on entry of X4 HIV-1 (pNL4.3) particles (**Fig. 7*A***). Similar to what has been reported previously, the IC_50_ of CXCL12 was approximately 1 nM. As a result of its binding to CXCR4, we reasoned that CXCL14 may also inhibit entry of X4 HIV-1 viruses or even synergize with CXCL12 in the prevention of HIV-1 entry into CD4^+^CXCR4^+^ target cells. To our surprise, however, treatment of TZM-bl cells with 10–1000 nM CXCL14 alone resulted in up to >3-fold enhancement of X4 HIV-1 infection (Fig. 7*B*). Furthermore, CXCL14 did not synergize with CXCL12 in inhibition of HIV-1 entry, as the suboptimal HIV-1 inhibition observed with 0.1 or 1 nM CXCL12 was not further potentiated. On the contrary, combination of the 2 chemokines resulted in a similar enhancement of HIV-1 entry as measured with CXCL14 alone (Fig. 7*B*). Inclusion of increasing concentrations of CXCL12 seemed to diminish the enhancement of X4 HIV-1 infection by CXCL14, which is in agreement with the critical role played by CXCR4 in this process. By using the same assay, we next examined the ability of CXCL14 to modulate the entry of R5-tropic HIV-1 (pR8Bal) particles. Pretreatment of TZM-bl cells, which coexpress CD4, CXCR4, and CCR5, with CXCL14 again led to a >3-fold increase in viral entry at the highest concentration tested (Fig. 7*C*). As expected (8, 9), CXCL12 was completely inactive in the prevention of R5 HIV-1 infection. The observed enhancement of R5 HIV-1 entry by CXCL14 was dependent on CXCR4, as infection of CXCR4-deficient GHOST cells that were engineered to coexpress CD4 and CCR5 by R5 HIV-1 was not affected by CXCL14 (Fig. 7*D*). We conclude that CXCL14 was unable to synergize with CXCL12 in the inhibition of HIV-1 infection, but, instead, substantially enhanced the infection of CD4^+^ target cells with both X4 and R5 HIV-1 particles.

## DISCUSSION

We report here an unexpected and strong synergistic cooperation between CXCL14 and CXCL12 in controlling chemokine functions in CXCR4^+^ immune cells. The importance of chemokines in the localization of immune cells is underscored by countless studies that have demonstrated a correlation between defects in chemokine expression (or function) and inflammatory diseases (1–3). CXCL14 belongs to the category of homeostatic chemokines as it is constitutively expressed in adult epithelial (nonlymphoid) tissues (14–21); therefore, it is remarkable that CXCL14 synergizes with CXCL12, another homeostatic chemokine with similar steady-state expression in adult peripheral tissues (60). As mentioned earlier, CXCL14 and CXCL12 are the most evolutionary conserved chemokines, and, in fact, are the only 2 chemokines with orthologs found in lower vertebrates (38). In addition, CXCL14 and CXCL12 show robust and complementary mRNA expression profiles during mouse and chicken embryogenesis (35–37), which suggests coordinated functions in embryonic tissue development. Indeed, deficiency in the expression of CXCL12 or its receptor CXCR4 is embryonically lethal (7). Similarly, we and others have reported previously that lack of CXCL14 results in a severe birth defect in mice (29–31). We hypothesize that the premature death of neonates in CXCL14-KO mice might be caused, in part, by the inability of CXCL12 to synergize with CXCL14.

Numerous reports have been published in recent years that describe functional synergism in the chemokine system, with several mechanisms, including chemokine heterodimerization, cell-surface clustering of chemokine receptors, and synergism in intracellular signaling pathways, put forward to explain this phenomenon (reviewed in refs. 51–53). Several reports have succeeded in demonstrating a positive effect of chemokine synergism in various *in vivo* models, including mouse models of monocyte recruitment to atherosclerotic lesions (61), neutrophil recruitment to the peritoneum (62), and rat models of leukocyte recruitment to inflamed skin (63) and the CNS (64). Similarly, we have recently reported that HMGB1, a chromatin-binding protein that is released by dying cells, was able to dimerize with CXCL12 and induce synergistic leukocyte recruitment in several mouse models of tissue necrosis (53). Furthermore, and more relevant to our current study, TGF-β family member activin A has recently been shown to induce DC migration *in vitro* as well as in an *ex vivo* model of chemokine-mediated emigration of DCs out of mouse ear tissue (25). Activin A by itself had no chemoattractant activity but induced in immature DCs the coordinate expression of CXCL14 and CXCL12 (but no other chemokine), with CXCL14 exceeding CXCL12 both in terms of kinetics and quantity. Of importance, immature DC migration was largely dependent on CXCR4, which suggests a mechanism similar to the one discussed here in our study with human blood lymphocytes and CXCR4^+^ cell lines.

**Figure 8.**
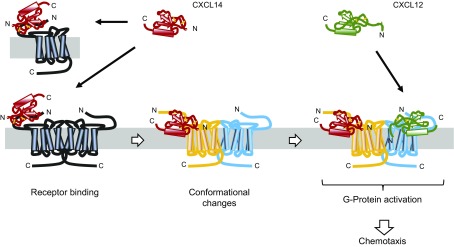
CXCL14 is a PAM of CXCR4. The model explains how CXCL14 is able to synergize with CXCL12 in the induction of CXCR4-mediated chemokine responses. The pool of cell-surface CXCR4 consists of a combination of individual CXCR4 conformation states, some of which are empty receptors in monomeric, dimeric, or oligomeric arrangements, whereas other conformational states are influenced by ligand binding (shown here by shift from black to yellow conformation upon CXCL14 binding). Binding of CXCL14 to CXCR4 by itself does not generate chemokine responses. Instead, CXCL14 binding induces allosteric changes in partner molecules that are present in CXCR4 homodimers or oligomers, thereby lowering the threshold of receptor activation by the functional ligand CXCL12 (shown here by shift from black to blue conformation in the partner molecule). As a result, subactive concentrations of CXCL12 become active, which leads to G-protein signaling and cellular responses.

Our findings support a new model of functional synergism among chemokines by proposing that CXCL14 acts as a positive allosteric modulator (PAM) for CXCR4 (**Fig. 8**) (65). This is in clear contrast to previous reports on chemokine synergism, which highlighted the ability of chemokines to form heterocomplexes or to act simultaneously by binding their respective chemokine receptors on the same target cell (reviewed in refs. 51, 52). Our negative NMR and SPR data exclude the possibility of CXCL14–CXCL12 heterodimer formation as an explanation for the observed functional synergy. In addition, and in clear contrast to blood monocytes (14, 15), blood lymphocytes and NK cells do not express functional receptors for CXCL14, which eliminates the possibility of 2 distinct chemokine receptors working in concert. Instead, and in agreement with previous results (32), our SPR experiments clearly showed that CXCL14 bound to CXCR4 with high affinity, which was comparable to what we have reported for CXCL12, the principal ligand for CXCR4 (44). CXCL14 is remarkable in its extremely short N-terminal sequence (Ser-Lys) that immediately precedes the first of 4 conserved Cys residues (Fig. 5). Of importance, short truncations in the N terminus of CXCL12 led to a complete loss of activity, which was in keeping with the 2-step model for chemokine–receptor interactions (55, 66, 67). Step 1 relates to the initial binding of the chemokine to its receptor and this step involves the globular core of chemokines, which includes the N loop that immediately follows the second Cys residue and regions defined by the 3 antiparallel β strands. Step 1 by itself does not lead to receptor activation. Step 2 involves the docking of the unordered N terminus of the chemokine to the minor and/or major binding pockets in the receptor, which leads to allosteric changes in the receptor and subsequent G-protein activation. Altogether, the high-affinity binding of CXCL14 to CXCR4 is remarkable and, on the basis of the missing N terminus in CXCL14, its inability to trigger CXCR4-mediated signaling events seems obvious. We propose that the observed synergy between CXCL14 and CXCL12 is not a result of to their simultaneous action on single-receptor molecules but, instead, involves both receptors in CXCR4 homodimers (or oligomers) as illustrated in Fig. 8.

GPCRs are known to coexist in multiple conformational states on cell surfaces, each state being associated with a distinct functional outcome (65). Each conformational intermediate (conformer) is defined by a loosely coupled allosteric network composed of an extracellular ligand binding site (step 2 binding pocket in chemokine receptors), connector domains, and intracellular effector (G proteins and β-arrestins) binding sites, each able to exist in various conformational states. Receptor conformations are also influenced by membrane context (membrane microdomains) as well as receptor clustering (receptor dimer/oligomerization) (68). It has been proposed that ligands select certain conformational states in GPCRs for binding and/or induction of signal transduction (49, 69); therefore, our model proposes that CXCL14, by binding to CXCR4, shifts the balance of CXCR4 conformers in favor of those recognized by the functional ligand CXCL12 (Fig. 8). CXCL14, by itself, is not an agonistic PAM (*i.e.,* the proposed conformational changes induced by CXCL14 binding were not sufficient for triggering chemokine responses in primary lymphocytes and CXCR4-transfected cell lines). Instead, the PAM activity of CXCL14 affects partner molecules in CXCR4 homodimers (or oligomers) by inducing conformational changes in neighboring (CXCL14-free) receptors (Fig. 8). Although CXCL14 displaced CXCR4-bound CXCL12, our experiments did not exclude the possibility that CXCL14-mediated conformational changes promoted changes in binding affinity between CXCL12 and its receptor. Our findings agree with a recent study that described high-affinity binding of CXCL14 to CXCR4 (32); however, PAM activity for CXCL14 was not reported. On the contrary, CXCL14 was either found to be inhibitory (32) or to lack inhibitory activity on CXCR4-expressing cells (34). The discrepancy of our study is not clear at present but may be explained in part by several factors, including the choice of target cells (monocytic cell line THP-1 cells, mouse pre–B cell line BaF/3, and CXCR4-transfected HEK293 epithelial cells), different combinations of chemokine (CXCL12 and CXCL14) concentrations, or different types of examinations that were carried out.

PAM activity of CXCL14 extends to the second natural ligand for CXCR4, gp120 of X4-tropic HIV-1. Here, we propose that CXCL14 stabilizes preferred conformations of CXCR4 for HIV-1 infection, possibly by promoting conformational changes in CXCR4 aggregates. The observed enhancement effect on HIV-1 infection was substantial (3- to 4-fold). Of note, CXCL14 did not synergize with CXCL12 in its HIV-1 suppressor activity (*i.e.,* CXCL14 did not increase the potency of CXCL12 to inhibit HIV-1 infection). Instead, it seems that CXCL14 synergized with gp120 in the viral entry response, irrespective of whether CXCL12 was present. Recent evidence suggests that chemokine-mediated receptor oligomerization is required for efficient chemokine responses (70). Although more experiments are needed to clarify the exact mechanism, it is possible that CXCL14-mediated CXCR4 clustering may facilitate gp120 binding and, thus, X4 HIV-1 infection. Surprisingly, CXCL14 had a similar enhancement effect on infection by R5-tropic HIV-1, which requires CCR5 as a coreceptor for entry into CD4^+^ target cells. CXCL14-mediated enhancement was dependent on the presence of CXCR4, which suggests that CXCL14 binding to CXCR4 had a positive effect on CCR5 conformers, possibly as a result of a shift in CCR5 conformers within CXCR4–CCR5 aggregates. We recently reported that CD4, CXCR4, and CCR5, when coexpressed simultaneously, form trimeric complexes that block HIV-1 binding and infection (58). It is thus plausible that CXCL14 binding to CXCR4 disrupts these trimeric receptor complexes, thereby facilitating the interaction of HIV-1 R5 viruses with CD4/CCR5. Regardless of the mode of action, it is noteworthy that CXCL14 is constitutively expressed up to very high levels in healthy mucosal tissues; therefore, local CXCL14 could adversely influence the course of HIV-1 infection by boosting the permissiveness in CD4^+^ target cells at sites that are critically involved in person-to-person HIV-1 transmission.

In summary, our model predicts that CXCL14 is a natural PAM for CXCR4, which, at present, is unique among chemokines and, to our knowledge, among physiologic ligands for other GPCRs (65). Our results will help to guide the design of allosteric modulators specific for CXCR4 to modulate tissue repair processes, cancer, or HIV-1 infection.

## AUTHOR CONTRIBUTIONS

P. J. Collins, M. L. McCully, S. Caucheteux, H. Lortat-Jacob, D. F. Legler, M. Ugguccioni, M. Thelen, M. Mellado, and B. Moser designed research; P. J. Collins, M. L. McCully, L. Martinez-Munõz, C. Santiago, J. Wheeldon, S. Thelen, V. Cecchinato, J. M. Laufer, V. Purvanov, and Y. R. Monneau performed research and analysed data; and P. J. Collins, M. L. McCully, S. Caucheteux, H. Lortat-Jacob, D. F. Legler, M. Ugguccioni, M. Thelen, M. Mellado, and B. Moser wrote the paper.

## Abbreviations

CCL: CC chemokine ligand
CCR: CC chemokine receptor
CFP: cyan fluorescent protein
CXCL: CXC chemokine ligand
CXCR: CXC chemokine receptor
DC: dendritic cell
FACS: fluorescence-activated cell sorting
FCS: fetal calf serum
FRET: Förster resonance energy transfer
LVP: lentiviral particle
PAM: positive allosteric modulator
PBMC: peripheral blood mononuclear cell
PE: phycoerythrin
SPR: surface plasmon resonance
YFP: yellow fluorescent protein
